# The HIV/AIDS epidemic in Cuba: description and tentative explanation of its low HIV prevalence

**DOI:** 10.1186/1471-2334-7-130

**Published:** 2007-11-09

**Authors:** Héctor de Arazoza, Jose Joanes, Rachid Lounes, Camille Legeai, Stéphan Clémençon, Jorge Pérez, Bertran Auvert

**Affiliations:** 1 Facultad de Matematica y Computacion, Universidad de La Habana, La Habana, Cuba; 2 Ministerio de Salud Publica, La Habana, Cuba; 3 Université René Descartes, Laboratoire MAP5, UMR-CNRS 8145, Paris, France; 4 Modalx – Université Paris X Nanterre; LPMA – UMR CNRS 7599 – Universités Paris 6 and Paris 7, France; 5 Instituto de Medicina Tropical «Pedro Kouri», La Habana, Cuba; 6 INSERM U687, 14 rue du Val d'Osne 94415 Saint-Maurice, France; 7 Hôpital Ambroise Paré, Boulogne-Billancourt, France; 8 Université de Versailles – Saint-Quentin; UFR médicale Paris-Ile-de-France-Ouest, Garches, France

## Abstract

**Background:**

The Cuban HIV/AIDS epidemic has the lowest prevalence rate of the Caribbean region. The objective of this paper is to give an overview of the HIV/AIDS epidemic in Cuba and to explore the reasons for this low prevalence.

**Methods:**

Data were obtained from the Cuban HIV/AIDS programme established in 1983. This programme has an extensive adult HIV testing policy, including testing of all pregnant women. HIV and AIDS cases have been recorded since 1986. Persons found to be HIV-positive are interviewed on their sexual behaviour and partners. Tracing and voluntary testing of these partners are organised. Epidemiological description of this epidemic was obtained from analysis of this data set. Using elementary mathematical analyses, we estimated the coverage of the detection system (percentage of HIV-positive adults detected) and the average period between HIV infection and detection. Estimated HIV prevalence rates were corrected to account for the coverage.

**Results:**

HIV prevalence has increased since 1996. In 2005, the prevalence among pregnant women was 1.2 per 10,000 (16/137000). Estimated HIV prevalence among 15- to 49-year-olds was 8.1 per 10,000 (4913/6065000; 95%CI: 7.9 per 10,000 – 8.3 per 10,000). Most (77%) of the HIV-positive adults were men, most (85.1%) of the detected HIV-positive men were reported as having sex with men (MSM), and most of the HIV-positive women reported having had sex with MSM. The average period between HIV infection and detection was estimated to be 2.1 years (IQR = 1.7 – 2.2 years). We estimated that, for the year 2005, 79.6% (IQR: 77.3 – 81.4%) of the HIV-positive persons were detected.

**Conclusion:**

MSM drive the HIV epidemic in Cuba. The extensive HIV testing policy may be an important factor in explaining the low HIV prevalence. To reduce the HIV epidemic in Cuba, the epidemic among MSM should be addressed. To understand this epidemic further, data on sexual behaviour should be collected. Now that antiretroviral therapy is more widely available, the Cuban policy, based on intensive HIV testing and tracing of partners, may be considered as a possible policy to control HIV/AIDS epidemics in other countries.

## Background

After sub-Saharan Africa, the Caribbean Basin has the second highest rate of HIV/AIDS in the world [1]. Cuba, an archipelago of this Caribbean Basin, comprises the island of Cuba and several smaller islands, with 11.2 million inhabitants (6.1 million in the age group 15–49). Life expectancy at birth is 75 and 80 years for men and women, respectively [2], the highest in Latin America.

The Joint United Nations Programme on HIV/AIDS (UNAIDS) in their reports on the global HIV/AIDS epidemic for 2005 and 2006 estimates that national adult HIV prevalence surpasses 1% in Barbados, Dominican Republic and Jamaica, 2% in Trinidad and Tobago, and exceeds 3% in Haiti and the Bahamas [3,4]. These reports indicate that the region's epidemics are driven primarily by heterosexual intercourse and estimate for Cuba an adult HIV prevalence of 0.1%. This is the lowest HIV prevalence in the Caribbean.

The objectives of this paper are to give an overview of the current HIV/AIDS epidemic in Cuba and to explore the reasons for its low prevalence.

## Methods

Data were obtained from the Cuban HIV/AIDS programme established in 1983 following developments of AIDS epidemics in other countries. The detection of the first HIV-positive person in Cuba took place in December 1985. This person was a heterosexual male returning from travel abroad. The first death due to AIDS occurred in April 1986 and was officially announced in the Cuban press soon after.

This Cuban HIV/AIDS programme included a system to detect HIV cases from several sources. Some of these sources started to be used at the beginning of the programme, others were introduced later and some have been discontinued. Since 1993, this detection system has focused on 6 major sources. These are blood donors, persons treated for other sexually transmitted infections, persons admitted to hospital with suspected HIV infection or subject to specific procedures like dialysis, persons volunteering to be tested, persons whose general practitioner has recommended HIV testing and, lastly, sexual partner tracing (see below). Other sources include testing of all pregnant women and prison inmates.

Since 1990, each time a person is tested for HIV, she/he is informed that such a test is going to be performed. When a person is found to be HIV-positive, the following information is collected and recorded in a national HIV/AIDS database: date of detection, age, gender, area of residence, gender of sexual partners in the past two years. A man having sex with men (MSM) is defined in this paper as a male reporting at least one sexual contact with another man in the past 2 years. From 1986, a person testing HIV-positive is interviewed by health workers using a non-anonymous structured questionnaire. They are invited to give names and contact details of sexual partners of the past two years. These partners are then traced and a recommendation for voluntary HIV testing is made [5].

Before 1994, persons who tested HIV-positive were followed up by the national health programme in AIDS sanatoria, which generated a lively and controversial debate [6-8]. This programme has evolved from its original conception and an outpatient care system was started in 1994 for those who wanted to leave the sanatoria. By the end of 2003, 60% of HIV-positive persons lived outside the sanatoria [9]. Each HIV-positive person receives, either in the sanatoria or in the outpatient system, regular counselling on living with HIV and how to prevent the risk of transmitting the virus.

AIDS cases are defined by the national health programme as HIV-positive persons with at least one opportunistic infection or, since the year 2000, a CD4 count of less than 200 cells/mm^3^. When a person is diagnosed with AIDS, the date of diagnosis is reported to the health authorities and added to the national HIV/AIDS database. Dates of death are also recorded in this database.

Antiretroviral therapy (ART) became available in 2000 and today every person with AIDS or every HIV-positive person with a CD4 count of less than 300 cells/mm^3 ^receives ART free of charge. Pregnant women are all tested for HIV and ART is proposed free of charge to pregnant women who test HIV-positive in order to reduce the risk of transmission of HIV to their child.

In this paper, we analyse HIV and AIDS cases recorded yearly in the national HIV-AIDS database. HIV prevalence among adults at the end of a year (y) was calculated as the number of cases of persons living with HIV. Prevalence rates among adults were calculated by dividing the number of HIV cases from the Cuban HIV/AIDS database by the adult population. Confidence intervals for prevalence rates were calculated assuming a binomial distribution.

In this paper, following the Cuban definition, adults were defined as persons aged 15 years or more. The annual prevalence rate among pregnant women was calculated as the number of pregnant women who have tested HIV-positive in one year divided by the total number of births during the same year.

The average interval (T) between HIV infection and detection was estimated by 2 methods. The first method assumes a steady state. In this case, T is calculated by dividing the number (P) of HIV-positive persons a year (y) whose HIV infection was not already known by the number (I) of HIV cases newly detected in the same year.

T=PI
 MathType@MTEF@5@5@+=feaafiart1ev1aaatCvAUfKttLearuWrP9MDH5MBPbIqV92AaeXatLxBI9gBaebbnrfifHhDYfgasaacPC6xNi=xI8qiVKYPFjYdHaVhbbf9v8qqaqFr0xc9vqFj0dXdbba91qpepeI8k8fiI+fsY=rqGqVepae9pg0db9vqaiVgFr0xfr=xfr=xc9adbaqaaeGacaGaaiaabeqaaeqabiWaaaGcbaGaemivaqLaeyypa0tcfa4aaSGaaeaacqWGqbauaeaacqWGjbqsaaaaaa@313C@

This calculation also assumes that AIDS mortality is negligible during the period T. This calculation is still valid when the relative change in HIV prevalence during T is small (quasi steady state). T was calculated using equation 1 and the data of the national HIV-AIDS database. The factor P was estimated as the number of pregnant women who test HIV-positive in the year y whose HIV infection was not already recorded by the detection system. The factor I was estimated in the year y by multiplying the annual number of HIV cases detected among women aged 15–49 by the proportion of pregnant women in the same group. These calculations were carried out year by year in the period 1996–2006. A mean and an interquartile range (IQR) were calculated from these values.

When P and I are a function of time (t), P(t) is given by the following equation:

P(t)=∫t−TtI(u)du
MathType@MTEF@5@5@+=feaafiart1ev1aaatCvAUfKttLearuWrP9MDH5MBPbIqV92AaeXatLxBI9gBaebbnrfifHhDYfgasaacPC6xNi=xI8qiVKYPFjYdHaVhbbf9v8qqaqFr0xc9vqFj0dXdbba91qpepeI8k8fiI+fsY=rqGqVepae9pg0db9vqaiVgFr0xfr=xfr=xc9adbaqaaeGacaGaaiaabeqaaeqabiWaaaGcbaGaemiuaaLaeiikaGIaemiDaqNaeiykaKIaeyypa0Zaa8qCaeaacqWGjbqscqGGOaakcqWG1bqDcqGGPaqkcqWGKbazcqWG1bqDaSqaaiabdsha0jabgkHiTiabdsfaubqaaiabdsha0bqdcqGHRiI8aaaa@3FE0@

When P(t) and I(T) are a linear function of time, this equation gives T as:

T=dPdtdIdt
 MathType@MTEF@5@5@+=feaafiart1ev1aaatCvAUfKttLearuWrP9MDH5MBPbIqV92AaeXatLxBI9gBaebbnrfifHhDYfgasaacPC6xNi=xI8qiVKYPFjYdHaVhbbf9v8qqaqFr0xc9vqFj0dXdbba91qpepeI8k8fiI+fsY=rqGqVepae9pg0db9vqaiVgFr0xfr=xfr=xc9adbaqaaeGacaGaaiaabeqaaeqabiWaaaGcbaGaemivaqLaeyypa0tcfa4aaSGaaeaadaWcaaqaaiabdsgaKjabdcfaqbqaaiabdsgaKjabdsha0baaaeaadaWcaaqaaiabdsgaKjabdMeajbqaaiabdsgaKjabdsha0baaaaaaaa@3982@

A second estimation of T was obtained using this equation 3 applied to the period 1996–2005 with dP/dt and dI/dt estimated by fitting a linear regression line through the data among pregnant women as described above in the first method. This second estimation is based on derivatives and, thus, has too wide a confidence interval to be indicated.

T0 being the incubation period, the coverage (c) can also be estimated by the following equation:

c=1−TT0
 MathType@MTEF@5@5@+=feaafiart1ev1aaatCvAUfKttLearuWrP9MDH5MBPbIqV92AaeXatLxBI9gBaebbnrfifHhDYfgasaacPC6xNi=xI8qiVKYPFjYdHaVhbbf9v8qqaqFr0xc9vqFj0dXdbba91qpepeI8k8fiI+fsY=rqGqVepae9pg0db9vqaiVgFr0xfr=xfr=xc9adbaqaaeGacaGaaiaabeqaaeqabiWaaaGcbaGaem4yamMaeyypa0JaeGymaeJaeyOeI0scfa4aaSGaaeaacqWGubavaeaacqWGubavcqaIWaamaaaaaa@3443@

Each year the detection system finds a fraction of the number of HIV-positive persons not yet detected. This fraction defined the performance (p) of the detection system. This performance was estimated, year by year, by dividing a number (n1) by a number (n2).

p=n1n2
 MathType@MTEF@5@5@+=feaafiart1ev1aaatCvAUfKttLearuWrP9MDH5MBPbIqV92AaeXatLxBI9gBaebbnrfifHhDYfgasaacPC6xNi=xI8qiVKYPFjYdHaVhbbf9v8qqaqFr0xc9vqFj0dXdbba91qpepeI8k8fiI+fsY=rqGqVepae9pg0db9vqaiVgFr0xfr=xfr=xc9adbaqaaeGacaGaaiaabeqaaeqabiWaaaGcbaGaemiCaaNaeyypa0tcfa4aaSGaaeaacqWGUbGBcqaIXaqmaeaacqWGUbGBcqaIYaGmaaaaaa@33DC@

Using these values, a mean and an IQR were calculated. In this equation, n1 is the number of HIV-positive women given in the year y by the detection system in the age group 15–49 weighted by the proportion of pregnant women among the women of the same age group in the same year. n2 is the number of HIV-positive women given by the detection system in the year y in the age group 15–49. This estimation is close to reality because all pregnant women are tested for HIV. The value of p calculated using equation 5 assumes that HIV prevalence among women in the age group 15–49 is close to the HIV prevalence among pregnant women of the same age.

We defined the coverage (c) of the detection system at a given time as the percentage of persons living with HIV that were detected before that time. This coverage was estimated in the following way. At the end of a year (y), from the data base we have the total number of cumulated detected HIV cases (H_0_) and the number of new detections in the same year (H_y_). Using the performance of the detection system, we estimated the total accumulated number of HIV infections (H_t_) as H_t _= H_0_+H_y_/p. The coverage of the detection system is then defined as the ratio H_0_/H_t_.

c=H0Ht
 MathType@MTEF@5@5@+=feaafiart1ev1aaatCvAUfKttLearuWrP9MDH5MBPbIqV92AaeXatLxBI9gBaebbnrfifHhDYfgasaacPC6xNi=xI8qiVKYPFjYdHaVhbbf9v8qqaqFr0xc9vqFj0dXdbba91qpepeI8k8fiI+fsY=rqGqVepae9pg0db9vqaiVgFr0xfr=xfr=xc9adbaqaaeGacaGaaiaabeqaaeqabiWaaaGcbaGaem4yamMaeyypa0tcfa4aaSGaaeaacqWGibascqaIWaamaeaacqWGibascqWG0baDaaaaaa@33A7@

A mean coverage was calculated using the mean performance. An estimation of the IQR of the coverage was calculated by using the IQR of the performance. To take into account the coverage of the detection system, a corrected value of HIV prevalence for men and women was calculated by dividing by c, given by equation 6, the prevalence given by the data base. We assumed that this coverage is not dependent on gender.

The median (IQR) interval between HIV detection and AIDS was calculated by survival analysis using the dates of HIV detection and the dates of AIDS using data prior to the introduction of ART in 2000.

This study has been approuved by the Ethics Committee of the Instituto de Medicina Tropical "Pedro Kouri", Havana Cuba in its monthly meetting of January 2005.

## Results

On average 1.6 million HIV tests were performed annually in the period 2000–2005. Table 1 shows the distribution of detected HIV cases according to how they were detected in the period 2000–2005. The HIV cases detected by sexual partner tracing or following a visit to a general practitioner represented 48.9% of the newly detected HIV cases in this period.

**Table 1:** Distribution of HIV cases detected according to how they were detected in the period 2000–2005

***How detected***	% of total
Test recommended by general practitioners	27.1
Sexual partner tracing	21.7
Voluntary testing	12.1
Blood donors	9.7
HIV test done in hospitals	8.9
Persons with other sexually transmitted infections	8.3
Pregnant women	1.6
Other	10.6
*Total*	100% (n = 4276)

**Figure 1:**
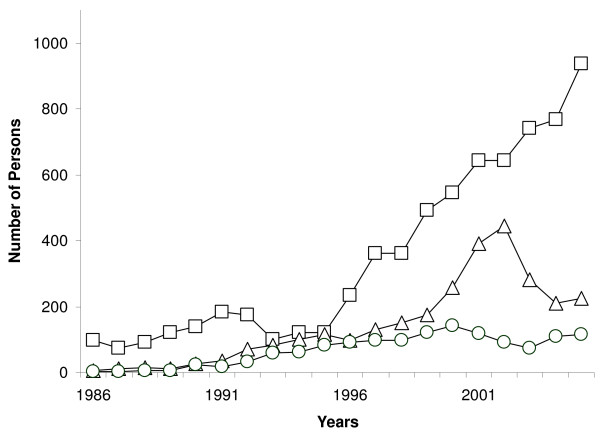
Number of detected HIV cases (square), AIDS cases (triangle) and deaths (circle) due to AIDS per year as given by the Cuban HIV/AIDS database.

Figure 1 shows the number of HIV infections, AIDS cases and deaths due to AIDS detected per year from 1986 to 2005. This figure shows that the number of HIV cases detected has increased sharply since 1996. The drop in HIV cases in the period 1993–1996 corresponds to a period of economic restrictions that had repercussions on the efficiency of the detection system. In 2005, 942 HIV infections were detected, with 887 (94.2%) among 15- to 49-year-olds. The increase in the number of AIDS cases detected from the year 2000 could be influenced by the introduction of ART, as patients that did not attend their scheduled consultations started attending them to obtain the newly available treatment, and at that time were diagnosed with AIDS.

Just before the introduction of ART, in the year 1999, 175 adult AIDS cases were recorded, among whom a high proportion of men (76.6%). Most of these 175 cases (140, 80.0%) had already been known to be HIV-positive. In the same year, the proportion of AIDS cases among women already known to be HIV-positive (37/41; 90.2%) was close to that among men (103/134; 76.9%, p = 0.075, Fisher exact test)

**Figure 2:**
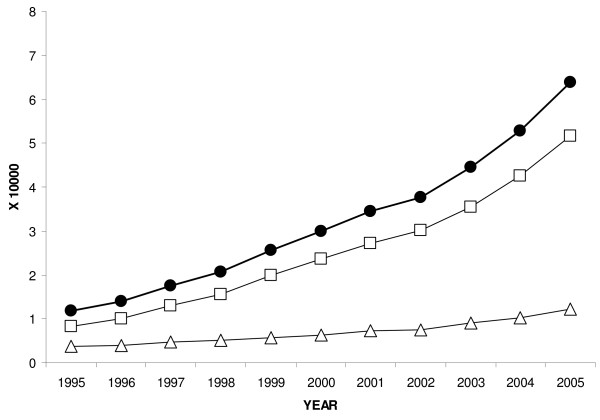
Uncorrected HIV prevalence among 15- to 49-year-old adults for all (circle), for women (triangles) and for men (squares).

Figure 2 shows the distribution of the HIV prevalence rate, not corrected for coverage, among adults and as a function of gender by year from 1995 to 2005. This figure shows a constant increase of HIV prevalence since 1995. In 2005, the uncorrected HIV prevalence was 6.4 per 10,000 (3866/6065000; 95%CI: 6.2 per 10,000 – 6.6 per 10,000).

In 2005, the female-to-male ratio among all adults was 1:5.2, so 83.8% of the HIV-positive adults were men. This percentage was quasi-constant from 2000 to 2004 with values of 82.7, 81.0, 86.2, 80.6 and 85.4%, respectively. In 2005, most (85.3%) of the men detected as HIV-positive were reported as being MSM.

In the period 2000–2005, interviews were carried out with 495 women who had been found to be HIV-positive and who represented 68.2% of those who tested HIV-positive. The sexual partners of these women were then interviewed as to their sexual preferences, and it was found that 70.9% of the women in the sample had sexual partners that were MSM.

**Figure 3:**
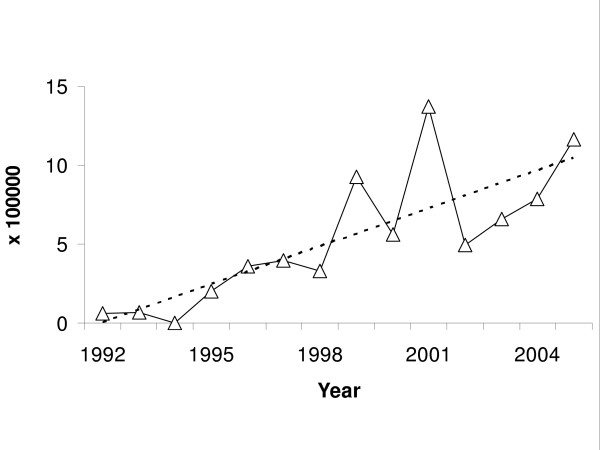
HIV prevalence rate in pregnant women as given by the Cuban HIV/AIDS database. The dotted line shows the linear trend.

Figure 3 shows the HIV prevalence rate among pregnant women by year from 1992 to 2005. This prevalence rate increases as a function of time. The slope of the linear regression line was 0.8% per year. In 2005, the HIV prevalence rate among pregnant women was 1.2 per 10,000 (16/137000).

Using equation 1, the average interval between HIV infection and detection was estimated to be 2.1 years (IQR = 1.7 – 2.2 years). Using equation 3, we found 2.5 years. Assuming an incubation period of 10 years, using equation 4, these two values lead to an estimated coverage of 0.79 and 0.75, respectively.

Using equation 5, the efficiency of the detection system for the period 1995–2005 was 52.9% (IQR: 46.3 – 59.2%). Using equation 6, the coverage of the detection system for the year 2005 was 79.6% (IQR: 77.3 – 81.4%), which is consistent with the high proportion of people already detected among AIDS cases (see above). It is also consistent with the values calculated using equation 4 (see above).

The corrected HIV prevalence rate in 2005 among 15- to 49-year-olds was 8.1 per 10,000 (4913/6065000; 95% CI: 7.9 per 10,000 – 8.3 per 10,000).

The median interval between detection and AIDS in the period 1995 – 2004 was 6.7 years (IQR = 6.1 – 7.2), which when added to the estimated time lapse between infection and detection (2.1 or 2.5 years) leads to an estimated incubation period of 8.8 years or 9.2 years.

In 2005, 88 million condoms were made available to the Cuban population through the retail pharmacy system. The 2004 figure was 82 million. Another 3 million condoms were distributed, free of charge, through educational and prevention programmes.

## Discussion

This paper shows that the HIV epidemic in Cuba is characterised by a low but rising HIV prevalence and by a majority of HIV cases among men having sex with men. These results indicate that HIV in Cuba is different from HIV epidemics in other Caribbean countries where the prevalence rates are higher and where the epidemics are driven primarily by heterosexual intercourse [3].

Several factors may have had some influence on the HIV epidemic data reported in this paper. The detection system has changed with time and was not fully functioning in the period 1993–1996. The impact of these factors is in all likelihood limited because most of the analyses presented here relate to the period 1996–2005.

Nevertheless, bias in the system of detection of HIV cases may also have led to incorrect estimation of the epidemic. We found this detection system's coverage to be high. Prevalence data calculated from the Cuban HIV/AIDS programme were corrected to take into account this coverage.

Several studies have used deterministic mathematical models to analyse the Cuban HIV/AIDS database and epidemic. Using data up to 1999, a first study reported an efficiency of 45.5% [10]. A second study, using data up to 2001, showed that the coverage is between 75% and 87%. [11]. A third study, using data up to 2002, estimated that it takes a mean 2.7 years to detect a person infected with HIV [12]. These estimations are consistent with the estimations given in this paper, which used the current version of the database and elementary epidemiological analyses.

Lastly, we cannot exclude a gender bias in the detection system, which could have led to a differential between coverage men and women. This differential could be due to a differential in reporting contact details of sexual partners by males and females when found to be HIV-positive. The impact of such a phenomenon is likely small because we found that the proportion of AIDS cases among persons already known as HIV-positive was as high among women as among men.

The low HIV prevalence rate in Cuba in comparison with other Caribbean countries is surprising. There is no reason to think that this is due to a late introduction of the virus because HIV was present in Cuba at the onset of the worldwide HIV epidemic. Two possible major explanations can be given for such a minor HIV epidemic: 1) Sexual behaviour may be different with less commercial sex and higher condom use, and 2) the intensive HIV testing policy with intensive counselling and follow-up of HIV-positive persons. More data on sexual behaviour in Cuba and in the other Caribbean countries are needed to assess the importance of the first possible explanation. The importance of the second possible explanation can be discussed qualitatively. The impact of the detection system in Cuba, which includes tracing of sexual partners, may have played an important role by allowing the detection of HIV-positive individuals only a couple of years after HIV acquisition, with a follow-up reducing the transmission of the virus to other persons.

## Conclusion

Cuba's approach to the HIV epidemic is specific and has been debated. Today, with the availability of ART, the ways chosen by Cuba to control the HIV/AIDS epidemic may be seen differently [13]. The intensive testing policy may be a useful approach to control an HIV epidemic, as a complement to other prevention approaches [14]. In addition, it should be emphasised that the information given to the population about the epidemic since the death of the first person in 1986 on TV, radio and other media may also have played a role in reducing the spread of HIV by alerting the population. Today, special programmes designed for teenagers have regular spots on HIV prevention and prevention of mother-to-child transmission of HIV is efficient: Cuba has a low percentage of vertical transmission (12%) [15]. The rising HIV prevalence is worrying. Despite current preventive and educational programmes carried out among MSM groups to advise them and to raise awareness about HIV acquisition and transmission, the clear increase in HIV among men shows that these programmes should be reinforced. Finally, more data, especially on sexual behaviour, are needed to analyse the Cuban HIV epidemic further and to gain insight into future trends in the epidemic.

## Competing interests

The author(s) declare that they have no competing interests.

## Authors' contributions

BA conceived of the study. HA and BA contributed equally to the writing of the paper. JJ gave access to data. RL, CL, SC and JP critically commented on the paper. All authors read and approved the final manuscript.

## Pre-publication history

The pre-publication history for this paper can be accessed here:



## References

[B1] Inciardi JA, Syvertsen JL, Surratt HL (2005). HIV/AIDS in the Caribbean Basin. AIDS Care.

[B2] WHO World Health Organisation: Cuba. Accessed: June 20, 2006.

[B3] UNAIDS-WHO AIDS epidemic update: December 2005.

[B4] UNAIDS 2006 Report on the global AIDS epidemic.. UNAIDS, Geneva 2006.

[B5] Hsieh YH, de Arazoza H, Lee SM, Chen CW (2002). Estimating the number of Cubans infected sexually by human immunodeficiency virus using contact tracing data. Int J Epidemiol.

[B6] Santana S, Faas L, Wald K (1991). Human immunodeficiency virus in Cuba: the public health response of a Third World country. Int J Health Serv.

[B7] Bayer R, Healton C (1989). Controlling AIDS in Cuba. The logic of quarantine. N Engl J Med.

[B8] Perez-Stable EJ (1991). Cuba's response to the HIV epidemic. Am J Public Health.

[B9] Pérez J, Pérez D, Gonzalez I, Diaz M, Orta M, Aragones C, Joanes J, Santin M, Lantero M, Torres R, Gonzalez A, Alvarez A (2004). Approaches to the management of HIV/AIDS in Cuba. Perspectives and management in antiretroviral treatment World Health Organization, Geneva.

[B10] Arazoza H, Lounes R, Hoang T, Interián Y (2000). Modeling the HIV Epidemic Under Contact Tracing - The Cuban Case. Journal of Theoretical Medicine.

[B11] de Arazoza H, Lounes R, Perez J, Hoang T (2003). What percentage of the Cuban HIV-AIDS epidemic is known?. Rev Cubana Med Trop.

[B12] de Arazoza H, Lounes R (2002). A non-linear model for a sexually transmitted disease with contact tracing. IMA J Math Appl Med Biol.

[B13] Susman E (2003). US could learn from Cuban AIDS policy. Aids.

[B14] De Cock KM, Marum E, Mbori-Ngacha D (2003). A serostatus-based approach to HIV/AIDS prevention and care in Africa. Lancet.

[B15] González I, Díaz M, Pérez J, Mengana H, Gutiérrez I, Gorry C (2006). National Program for Detecting & Treating Mother-to-Child Transmission of HIV. MEDICC Review.

